# Determining an anthropometric surrogate measure for identifying low birth weight babies in Uganda: a hospital-based cross sectional study

**DOI:** 10.1186/1471-2431-13-54

**Published:** 2013-04-12

**Authors:** Nabiwemba L Elizabeth, Orach Garimoi Christopher, Kolsteren Patrick

**Affiliations:** 1 School of Public Health, Makerere University College of Health Sciences, P. O. Box 7072, Kampala, Uganda; 2 Institute of Tropical Medicine, Nationalstraat 155, Antwerp, B-2000, Belgium

**Keywords:** Low birth weight anthropometric measurements predictor

## Abstract

**Background:**

Achieving Millennium Development Goal 4 is dependent on significantly reducing neonatal mortality. Low birth weight is an underlying factor in most neonatal deaths. In developing countries the missed opportunity for providing life saving care is mainly a result of failure to identify low birth weight newborns. This study aimed at identifying a reliable anthropometric measurement for screening low birth weight and determining an operational cut-off point in the Uganda setting. This simple measurement is required because of lack of weighing scales in the community, and sometimes in the health facilities.

**Methods:**

This was a hospital-based cross-sectional study. Two midwives weighed 706 newborns and measured their foot length, head, chest, thigh and mid-upper arm circumferences within 24 hours after birth.

Data was analysed using STATA version 10.0. Correlation with birth weight using Pearson’s correlation coefficient and Receiver Operating Characteristics curve analysis were done to determine the measure that best predicts birth weight. Sensitivity and specificity were calculated for a range of measures to obtain operational cut-off points; and Likelihood Ratios and Diagnostic Odds Ratio were determined for each cut-off point.

**Results:**

Birth weights ranged from 1370–5350 grams with a mean of 3050 grams (SD 0.53) and 85 (12%) babies weighed less than 2500 grams. All anthropometric measurements had a positive correlation with birth weight, with foot length showing the strongest (r = 0.76) and thigh circumference the weakest (r = 0.62) correlations. Foot length had the highest predictive value for low birth weight (AUC = 0.97) followed by mid-upper arm circumference (AUC = 0.94). Foot length and chest circumference had the highest sensitivity (94%) and specificity (90%) respectively for screening low birth weight babies at the selected cut-off points. Chest circumference had a significantly higher positive likelihood ratio (8.7) than any other measure, and foot length had the lowest negative likelihood ratio. Chest circumference and foot length had diagnostic odds ratios of 97% and 77% respectively. Foot length was easier to measure and it involved minimal exposure of the baby to cold. A cut-off of foot length 7.9 cm had sensitivity of 94% and specificity of 83% for predicting low birth weight.

**Conclusions:**

This study suggests foot length as the most appropriate predictor for low birth weight in comparison to chest, head, mid-upper arm and thigh circumference in the Uganda setting. Use of low cost and easy to use tools to identify low birth weight babies by village health teams could support community efforts to save newborns.

## Background

Globally neonatal mortality is 23/1,000 live births and it contributes to 60% and 40% of infant and under 5 mortality respectively. A third of all neonatal deaths occur in Sub-Saharan Africa, where neonatal mortality rate (NMR) is 35/1000 live births. While infant and child mortality have decreased over the last 20 years, neonatal mortality has almost remained unchanged and this has resulted in an increase in the proportion of infant and child deaths that occur in the neonatal period [1]. According to the Uganda Demographic and Health Survey 2011 preliminary report, NMR decreased from 36 to 27/1000 live births over the last 10 years but the proportion of infants that die during the neonatal period increased from 37% to 50% in the same period (UBOS & MEASURE). These trends in NMR indicate that Millennium Development Goal 4 will not be achieved unless deliberate efforts are directed towards reducing deaths during the neonatal period. The leading causes of neonatal mortality are complications of preterm births, intra-partum related complications and infections namely sepsis and pneumonia [2].

Low birth weight (LBW), defined as a weight less than 2500 grams, is an underlying factor in up to 70% of neonatal deaths in developing countries [3]. Low birth weight is associated with prematurity, high risk of infections, difficult breathing, hypothermia and feeding problems. Jitta and Kyaddondo estimated a prevalence of LBW in Uganda of 16% among babies that were weighed at birth [4]. Many LBW babies remain undetected because they are either born at home or due to logistical problems like non-availability of weighing scales, they are not weighed and are therefore deprived of the much needed care. It is imperative to identify these high risk babies early and give them adequate care needed for their survival. Deaths among LBW could be reduced with low cost interventions that focus on keeping the baby warm, hygiene, breast feeding support, early identification and management of illness in the first days and weeks of life [5,6]. There are efforts to improve survival of newborns through home visits by community health workers (CHWs) who are trained to identify and provide care for at risk newborns [7]. However, in Uganda these volunteers do not carry weighing scales as they are not readily available. No studies have been done in Uganda to identify alternative low cost methods for identifying high risk LBW babies at the community level.

Studies have been conducted in various settings to identify anthropometric surrogates for low birth weight. The anthropometric measurements that have been studied include; body length, foot length, head, chest, thigh, calf and mid-upper arm circumference [8-12]. Generally studies show that choice of a suitable anthropometric measure is context-specific. Various studies recommend using specific measures including head circumference [9], chest circumference [8,13], mid upper arm circumference [14,15], thigh circumference [10,16], foot length [17] and calf circumference [12,18] as the most appropriate. Even where the same measure was recommended the cut-off points varied for different contexts e.g. cut-off for identifying LBW using chest circumference was 30.5 cm in Bangladesh compared to 31.3 cm in Iran [8,19]. There is therefore need for a study to identify the most appropriate anthropometric surrogate for LBW and its cut-off in Uganda. To date there is no documentation of similar studies conducted in Uganda. The aim of this study was to identify the most reliable anthropometric measurements for screening low birth weight babies in the community and to determine the operational cut-off point for this measure in this setting. Here we report the appropriate predictor of low birth weight and the cut-off point.

## Methods

### Study setting

The study was carried out in Iganga-Mayuge Health and Demographic Surveillance Site, Eastern Uganda. Iganga and Mayuge Districts are predominantly rural, with 70% of the population living within five kilometres of a health facility. The area is served by one hospital and several lower level health centres. Preliminary results of the Uganda Demographic and Health Survey 2010/11 indicate that neonatal and infant mortality rates are 22.3 and 77.5 per 1000 live births respectively in the Eastern region of Uganda.

### Study design, data collection and analysis

**Figure 1:**
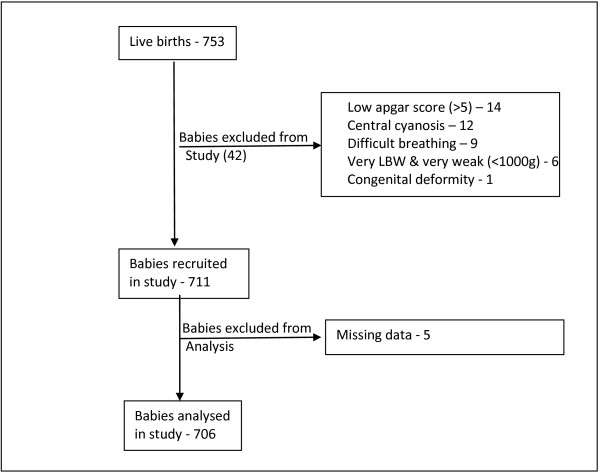
Flow chart showing number of newborns recruited, number excluded and reason for exclusion.

This was a hospital-based cross-sectional study carried out in Iganga General Hospital. Two hospital-based midwives and one doctor (clinical supervisor) were trained by the principal investigator for three days, on how to measure the newborns’ foot length, head, chest, thigh and mid-upper arm circumferences. Informed written consent was obtained from the mothers before their babies were measured. A sample size of 512 was obtained using Buderer’s formula with the following assumptions: sensitivity – 80%, LBW prevalence – 12% (Iganga Hospital data), absolute precision of sensitivity – 10% and α – 5% [20]. Data were collected between 1st September and 17th December 2009 by two midwives who assessed all live born babies for inclusion into the study. Babies with poor health conditions were excluded from the study in order to allow them get emergency care (Figure 1).

For each recruited baby, the following measurements were done within 24 hours after birth: 1. Head circumference was measured between the glabella anteriorly and along the occipital prominence at the back of the head; 2. mid-upper arm circumference (MUAC) was measured at the midpoint between the tip of the acromion process and olecranon process of the left upper arm; 3. chest circumference was measured at the nipple level at the end phase of expiration; 4. thigh circumference was measured at the lowest furrow of the gluteal region using a flexible non-elastic measuring tape; 5. foot length was measured from the heel to tip of the big toe using hard transparent plastic ruler.

The measurements were taken in centimetres to one decimal place. Birth weight was measured with a digital salter scale to the nearest 10 grams. The scale was calibrated before each use using a bottle weighing 1000 grams. The midwives and a clinical supervisor, who is a medical doctor, were trained for three days on how to take the measurements. For each measurement, two readings were done by one midwife and the average was taken. The clinical supervisor performed two repeat measurements for each test for 1 in every 30 study participants to check for reliability. Data were analysed using STATA version 10.0. Five babies with missing data were excluded from the analysis. For each anthropometric measure, the mean, standard deviation and difference between the means of normal weight and LBW babies were calculated using Student’s *t*-test. Correlation with birth weight was determined using Pearson’s correlation coefficient. Receiver operating characteristics (ROC) analysis was conducted separately for each measure and the area under the curve (AUC) calculated to explore which measure best predicted birth weight. Sensitivity and specificity were calculated for a range of measures to obtain operational cut-offs that could be used to identify small babies in the community. This operational cut-off was taken as the value with the highest average value for sensitivity plus specificity i.e. the lowest total misclassification error rate. Positive likelihood ratio (+LR), negative likelihood ratio (-LR) and diagnostic odds ratio (DOR) were determined at each cut-off point. This study was approved by Makerere University School of Public Health Institutional Review Board and Uganda National Council of Science and Technology.

## Results

During the 15 weeks period, there were 753 live births, of which 711 (94%) newborns were recruited into the study. Five babies had missing data, so analysis was done for data collected from 706 babies.

### Descriptive characteristics

Three hundred and eighty babies (54%) were male. The birth weights ranged from 1370–5350 grams with a mean of 3050 grams (SD 0.53) with 85 (12%) babies having a birth weight less than 2500 grams.

In addition to measurements taken by the midwife, repeat measurements were taken by the supervisor in 24 cases. Inter-observer agreement was tested using the Kappa statistic and the results were as follows; weight-0.46, HC-0.34, FL-0.30, MUAC-0.28, CC-0.22 and TC 0.18 and p < 0.01.

There was a significant difference in means of the normal weight and LBW groups for all measurements as shown by Students *t*-test (p < 0.001) [Table 1].

**Table 1:** Means and standard deviations of the different measurements

**Measure in cm**	**Range**	**Mean (SD)**			**Mean difference p-value**
		**All babies**	**Birth weight**	**Birth weight**	
			**< 2500 grams**	**≥ 2500 grams**	
**Head circumference (HC)**	23.5–54.5	34.4 (2.0)	32.0 (1.52)	34.7 (1.84)	0.0010
**Foot length (FL)**	6.0–9.3	7.9 (0.51)	7.2 (0.46)	8.1 (0.43)	0.0001
**Mid upper arm circumference (MUAC)**	6.0–19.0	10.9 (1.36)	9.1 (0.83)	11.1 (1.23)	0.0001
**Thigh circumference (TC)**	7.5–36.5	16.8 (2.27)	14.1 (1.35)	17.2 (2.11)	0.0010
**Chest circumference (CC)**	13.5–52.0	32.5 (2.64)	28.6 (2.14)	33.0 (2.23)	0.0006
**Weight in grams**	1370–5350	3050 (0.53)	2120 (0.27)	3280 (0.42)	0.0001

### Correlation coefficients and ROC curve analysis

**Figure 2:**
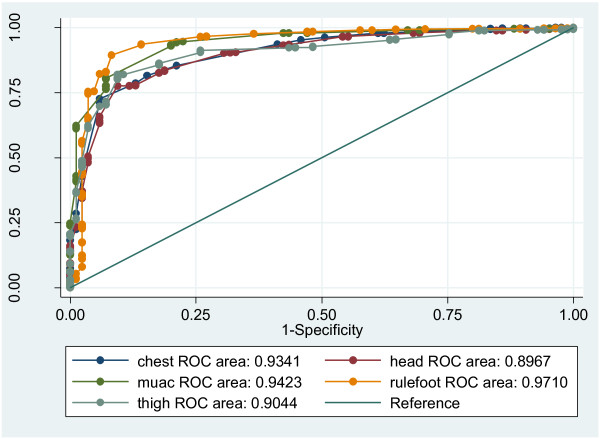
ROC curves for LBW and all anthropometric measurements.

All the anthropometric measurements were correlated with birth weight, with Pearson correlation coefficients between 0.62 and 0.76, which are categorised as moderate to strong correlations according to Dancey and Reidy’s categorisation [21]. The correlation coefficients(r) and coefficients of determination (r^2^) for the various measurements are shown in Table 2. ROC analysis to test how well the different measures predict LBW showed that foot length had the highest AUC (0.97) while HC had the lowest (0.89) [Figure 2]. AUC of FL was significantly higher than HC and TL, but not significantly different from CC and MUAC (Table 2).

**Table 2:** Correlation of anthropometric measurements with birth weight

	**AUC (95% CI)**	**Correlation coefficient, r (95% CI)**	**r**^**2**^
**Head circumference (HC)**	0.89 (0.86–0.93)	0.63 (0.54–0.67)	0.40
**Foot length (FL)**	0.97 (0.95–0.99)	0.76 (0.62–0.85)	0.58
**Mid upper arm circumference (MUAC)**	0.94 (0.92–0.97)	0.67 (0.58–0.74)	0.45
**Thigh circumference (TC)**	0.90 (0.87–0.93)	0.62 (0.51–0.65)	0.38
**Chest circumference (CC)**	0.93 (0.91–0.96)	0.72 (0.57–0.81)	0.52

### Sensitivity, specificity and likelihood ratios

For each anthropometric measure, sensitivity and specificity for the whole range of values were calculated and operational cut-off points determined by taking the value with the highest average of sensitivity and specificity. At the proposed cut-offs, FL and CC had the highest sensitivity (94%) and specificity (90%) respectively for screening LBW babies. Sensitivity of FL, MUAC, TC and CC were not significantly different except for HC which was significantly lower. Specificity of CC was significantly higher than for all other measurements (Table 3). Likelihood ratios and diagnostic odds ratios for all measures are shown in Table 4. CC had a significantly higher + LR than any other measurement. FL had the lowest –LR, but it was not significantly different from –LR of MUAC and CC. DOR was highest for CC (97.1) followed by FL (77.3).

**Table 3:** Sensitivity and specificity of the different anthropometric measurements for predicting LBW at selected cut-off points

	**(cm)**	**Birth weight**	**Birth weight**	**Sensitivity**	**Specificity**
		**< 2500 grams**	**≥ 2500 grams**	**(95% CI)**	**(95% CI)**
**Head circumference (HC)**	<33.3	70	109	82.4	82.4
	≥33.3	15	512	(79.4–85.5)	(72.6–89.8)
**Foot length (FL)**	<7.9	80	108	94.1	82.6
	≥7.9	5	513	(86.8–98.1)	(79.8–86.1)
**Mid upper arm circumference (MUAC)**	<10.1	79	121	92.9	80.5
	≥10.1	6	500	(85.3–97.4)	(77.2–83.6)
**Thigh circumference (TC)**	<15.7	77	113	90.6	81.8
	≥15.7	8	508	(82.3–95.8)	(78.7–84.9)
**Chest circumference (CC)**	<31.0	78	65	91.8	89.5
	≥31.0	7	556	(83.8–96.6)	(86.9–91.8)

**Table 4:** Likelihood ratios and diagnostic odds ratio for the different anthropometric measurements for predicting LBW at selected cut-off points

	**Cut-off (cm)**	**Positive likelihood ratio (95% CI)**	**Negative likelihood ratio (95% CI)**	**Diagnostic odds ratio (+LR/-LR)**
**Head circumference (HC)**	33.3	4.68	0.21	22.4
		(3.88–5.7)	(0.03–0.34)	
**Foot length (FL)**	7.9	5.41	0.07	77.3
		(4.52–6.47)	(0.03–0.17)	
**Mid upper arm circumference (MUAC)**	10.1	4.76	0.09	52.9
		(4.02–5.65)	(0.04–0.19)	
**Thigh circumference (TC)**	15.7	4.98	0.11	45.3
		(4.16–5.96)	(0.06–0.22)	
**Chest circumference (CC)**	31.0	8.71	0.09	97.1
		(6.89–11.0)	(0.04–0.19)	

## Discussion

This study shows that LBW newborns in Uganda can be identified using anthropometric surrogate measures on the first day of life. In resource poor settings where neonatal mortality remains high and many births occur at home, the missed opportunities for either providing life saving care at home or referral are mainly a result of a failure to identify high risk LBW newborns. Anthropometric measurements have been found to be reliable in identifying LBW. However, studies show that the measure of choice and its cut-off point is dependent on the context [10,14-16], therefore the need to conduct area specific studies. Previous studies show that more work has been done for other anthropometric measurements like HC, CC, TC and MUAC than for foot length as a predictor of LBW. This is one of the few studies that compares FL with other measurements.

Although in this study all the measurements could identify LBW to some degree, FL had a fairly strong correlation with birth weight (r = 0.76 and AUC = 0.97) although from the value of r^2^, only 58% of the variation in foot length can be explained by the linear relationship between FL and birth weight. Other analysis (sensitivity, specificity, +LR, -LR and DOR) indicate that the two most appropriate measurements for identifying LBW in Uganda are FL and CC.

A foot length cut-off of 7.9cm has a sensitivity of 94% and specificity of 83% for predicting LBW. At a cut-off of 7.9 cm the probability of a baby being low birth weight increases from 12% to 66% when the foot length is less than 7.9 cm (Table 3). Using a more practical cut-off of 8.0 cm for foot length increases the post-test probability from 12% to 64%.

The operational cut-off determined by our study for the different measures are comparable to cut-offs obtained in similar studies in some settings. For instance the cut-off for CC was 31.0 cm in this study which is comparable to the cut-off in Nepal (30.8 cm) and in Iran (31.2 cm) [8,9]; and that for HC was 33.3 cm in comparison to 33.5 cm in Nepal [9]. In this study foot length cut-off of 7.9 cm is comparable to 8.0 cm that was proposed by Marchant et al. in Tanzania [17].

On observation during the data collection, the midwives were finding it difficult to determine the exact point to measure the thigh, head and mid upper arm circumferences. Also timing the end of expiration to measure the chest circumference was challenging. This could have implications for use of these measurements by community health workers. However, foot length was practically easier and faster to measure. The ease with which foot length was measured compared to the rest makes it a preferred measure. A problem of accuracy of HC measurements due to moulding of the head especially when labour was prolonged or obstructed was noted by Dhar et al. [19]. Another advantage is that measuring the foot does not require undressing the baby thus exposing them to cold.

Although in this study, we did not test the usefulness of these measures after day 1 in identifying LBW, Marchant et al. demonstrated that foot length was a good predictor of LBW up to day 5 after birth [17]. This would be critical because in some cases the CHWs do not visit the newborns on the first day. A study in Uganda also showed that HC and CC can be measured in the first 2 weeks of life and extrapolated to estimate the measurement at day of birth [22].

Uganda has launched the Village Health Team concept which is compatible with the WHO and UNICEF recommendation to provide extra care for newborns at home [7]. It involves community health workers visiting homes to promote health, but provides no access to weighing scales in part because of cost and fears about their maintenance and sustainability. A low tech, low cost and low risk tool to identify small babies could support community efforts to save newborn lives if it is added to the VHT resources used during postpartum visits.

In using such a tool, we would be more concerned about minimising numbers of false negatives than false positives. This study tested HC, FL, MUAC, TC and CC, and showed that this is well achieved by FL. The potential risk of including babies that are falsely labelled LBW is minimal compared to the consequences of leaving out truly LBW babies.

### Limitations

This being a hospital-based study, the estimates of LBW may not reflect what is in the community. The measurements were done by health workers but the tool will be used by community volunteers and their skills are likely to be different. However a validation of this tool will be done in the community with community volunteers. There was potential for bias by having the same midwife perform both gold standard and index measurements, but this was minimised by the investigator not knowing the cut-off points for the index measurements. The exclusion of very small babies could have led to under estimation of the true sensitivity and specificity since these are more likely to be observed by the proxy measurement.

## Conclusion

This study suggests that foot length is the most appropriate predictor for LBW given its high predictive values and ease of measurement in comparison to head, chest, thigh and mid-upper arm circumference. A FL cut-off of 7.9 cm had the lowest total misclassification error rate.

Further research to establish how reliable foot length is when applied by non-medical persons e.g. community health workers and mothers themselves as compared to trained medical staff, as well as its acceptability is recommended. We also recommend further research to prospectively evaluate the foot length cut-off of 7.9 cm to screen LBW babies. Since some babies may not be visited on day one, studies should be done to determine the number of days after birth within which foot length remains useful for predicting LBW.

## Competing interests

The authors declare that they have no competing interests.

## Authors’ contributions

NLE was responsible for the conception and design of the study, statistical analysis and interpretation, and drafting the manuscript. OGC contributed to the conception and design of the study, statistical analysis, interpretation, reviewing and revising the manuscript. KP contributed to statistical analysis and interpretation, and reviewing and revising the manuscript. All authors read and approved the final manuscript.

## Authors’ information

NLE is a medical doctor with a Master of Medicine in Public Health. She is currently pursuing a PhD in child health with emphasis on low birth weight and newborn care practices in the community. She is a lecturer at the School of Public Health, Makerere University College of Health Sciences; and her areas of interest are maternal and child health, community health and health promotion.

OGC is medical doctor with a doctorate in public health. He is Associate Professor and Head of the department of community health and behavioural sciences at the School of Public Health, Makerere University College of Health Sciences. He teaches maternal and child health and reproductive health including reproductive health care services organisation for populations living in emergency settings.

KP is a medical doctor and holds postgraduate degrees in pediatrics, tropical medicine and a PhD in nutrition. He is a Professor at the Institute of Tropical Medicine, Antwerp, Belgium. He works in the field of child health and nutrition.

## Pre-publication history

The pre-publication history for this paper can be accessed here:

http://www.biomedcentral.com/1471-2431/13/54/prepub
